# *Spondias mombin* as a reservoir of fruit fly parasitoid populations in the Eastern Amazon: an undervalued ecosystem service

**DOI:** 10.7717/peerj.11530

**Published:** 2021-06-04

**Authors:** Maria do Socorro Miranda de Sousa, Ezequiel de Deus, Adilson Lopes Lima, Cristiane Ramos de Jesus, Salustiano Vilar da Costa Neto, Lailson do Nascimento Lemos, Ana Claudia Mendes Malhado, Richard J. Ladle, Ricardo Adaime

**Affiliations:** 1 Programa de Pós-graduação em Biodiversidade Tropical, Universidade Federal do Amapá, Macapá, Amapá, Brazil; 2 Instituto Federal do Amapá, Laranjal do Jari, Amapá, Brazil; 3 Embrapa Amapá, Macapá, Amapá, Brazil; 4 Instituto de Pesquisas Científicas e Tecnológicas do Estado do Amapá, Macapá, Amapá, Brazil; 5 Universidade Federal do Amapá, Mazagão, Amapá, Brazil; 6 Instituto de Ciências Biológicas e da Saúde, Universidade Federal de Alagoas, Maceió, Alagoas, Brazil; 7 Centro de Investigação em Biodiversidade e Recursos Genéticos, Universidade do Porto, Vairão, Portugal

**Keywords:** Biocontrol, Braconidae, Pest, Taperebá, *Anastrepha obliqua*, *Doryctobracon areolatus*, *Opius bellus*

## Abstract

Fruit flies are economically important pests that infest a wide variety of host trees. The environmental damage caused by traditional pesticide-based control methods has prompted scientists to seek less damaging alternatives such as biological control by native species. Parasitoids, especially Braconidae species, have excellent potential as biological control agents for fruit flies, being both generalists and well distributed geographically. Native fruit trees that support medium or high levels of these parasitoids could therefore play an important role in biological control strategies. A good potential example is *Spondias mombin* L. in the Brazilian Amazon, which hosts several species of fruit flies and associated parasitoids. Here, we provide a unique synthesis of over nearly two decades of data from the east Amazon, clearly demonstrating the potential of *S. mombin* to act as a source and reservoir of fruit fly parasitoids. This important ecosystem service (biological control) provided by the parasitoids and supported by *S. mombin* could be further enhanced through conservation of this plant species in its natural environment.

## Introduction

Biological control is recognized as an important regulating ecosystem service (ES) provided by biodiversity (Millennium Ecosystem Assessment, 2005). Understanding the factors that govern biological control in nature is essential for its successful application in agricultural systems. For example, the control of pests and insect vectors of pathogens by natural enemies such as predators, parasitoids and microorganisms (Sujii et al., 2020). Adopting strategies based on natural biological control may also lead to reduced use of chemical pesticides, reducing the exposure of rural workers to dangerous substances, decreasing the development of resistant strains of pests and minimizing contamination of the food produced (Hajek & St Leger, 1994; Bianchi, Booij & Tscharntke, 2006). However, the simplification of landscapes due to the adoption of intensive agriculture has caused significant declines in biodiversity with the associated loss of ecosystem services, including biological control (Bianchi, Booij & Tscharntke, 2006). Providing high levels of this service requires the maintenance of complex landscapes that can sustain populations of diverse natural enemies of target pest species. A greater diversity of plants in the landscape can, for example, provide stable food sources optimizing biological control in agroforestry systems (Landis, Wratten & Gurr, 2000; Rezende et al., 2014). In addition, greater plant richness can lower rates of attack by herbivorous arthropods and sustain a greater abundance of natural enemies (Andow, 1991).

A good example of the benefits of landscapes with high plant diversity is the case of Kelly’s citrus thrip *Pezothrips kellyanus* (Bagnall), which does not cause significant damage to citrus fruits when cultivated in soils with dense vegetation cover. This is because such soils contain high densities of mesostigmatid mites, a generalist predator of the thrips (Colloff, Elizabeth & Cook, 2013). Likewise, biological control of whitefly *Bemisia tabaci* was found to be more efficient on farms with more diverse vegetation (Togni et al., 2019). In both cases habitat quality was strongly linked to control effectiveness, and the ecosystem service can be considered as the result of the joint action of natural and human capital (details in Bengtsson, 2015). However, knowledge about the role of natural vegetation in maintaining populations of natural enemies is still limited and requires greater scientific input, especially in megadiverse countries, such as Brazil. More generally, there have been few studies on ecosystem services provided by insects in tropical regions (Noriega et al., 2018) including the vast expanse of the Amazon basin (Ramos et al., 2020).

Fruit flies (Diptera: Tephritidae) are among the best known pest species and have been extensively studied in the tropics due to the damage they cause to number of economically important cultivated species (Aluja, 1999; Uramoto, 2007; Aluja & Mangan, 2008). Some studies have indicated that larval infestation by fruit fly species may sometimes benefit host plant fitness (Drew, 1987) by facilitating seed dispersal and by accelerating the decomposition process (allowing faster seed germination). Wilson et al. (2012) tested this hypothesis, demonstrating that *Bactrocera tryoni* (Froggatt) larvae feeding accelerates the decomposition of the fruits and does not affect the number of seeds or their germination. They also demonstrated that, for some plant species, native rodents prefer infested fruits and concluded that infestation by fruit fly larvae has neutral or beneficial impacts on the host plant. These results suggest that fruit flies can provide a valuable support service for native vegetation. Most native parasitoids of the fruit fly genus *Anastrepha* are generalists (attacking many species of *Anastrepha*) and many native parasitoid species preferentially infest larvae of *Anastrepha* on native wild fruit trees. In other words, these parasitoids not only visit many host plant species used by *Anastrepha*, they also attack the larvae of many *Anastrepha* species (López, Aluja & Sivinski, 1999). Therefore, the interactions of fruit flies/parasitoids/host plants in native forests directly contributes to the conservation of the biological control ES.

Native vegetation is an important source of fruit fly parasitoids, and it has been repeatedly shown that wild native plants harbor significantly more parasitoids per fruit than cultivated plants (Sivinski, 1991; Hernández-Ortíz, Pérez-Alonso & Wharton, 1994; Aluja, 1999; López, Aluja & Sivinski, 1999). Given these findings, conserving or cultivating wild host plants near to commercial crops could increase the provision of biological control of fruit flies provided by parasitoids in agricultural environments (Aluja, 1994; Aluja, 1999; Newton et al., 2009). Such a strategy aligns well with biodiversity conservation (Millennium Ecosystem Assessment, 2005) and is compatible with other sustainable practices such as ecotourism.

Although Brazil is a large, megadiverse country, with significant biological diversity, scientific knowledge about the importance of insects in the provision of ecosystem services is very limited. Most of the existing knowledge is related to agricultural production, especially in the Atlantic Forest or the Cerrado biomes (Ramos et al., 2020). Fruit flies have been little studied in areas of native forests even though such studies are essential to understand the tritrophic interactions between fruit flies, their host plants and associated parasitoids (Jesus-Barros et al., 2012). Such research is urgently needed because the rapid deforestation of the tropics may be causing the disappearance or even the extinction of many species of fruit flies, consequently threatening the survival of associated parasitoid species (Aluja, 1999; Aluja et al., 2003).

The study of wild native plants that act as ‘reservoirs’ of fruit fly parasitoids has received growing interest in recent years, especially in the state of Amapá, Brazil, located in the extreme north of Eastern Amazonia. Adaime et al. (2018) studied the pioneer species *Bellucia grossularioides* (L.) Triana (Melastomataceae). The fruits were infested by *Anastrepha coronilli* Carrejo & González and four species of parasitoids were recorded (the mean percentage of parasitism was 12.8%). The authors concluded that *B. grossularioides* acts as a reservoir of fruit fly parasitoids and that the plant should be conserved where it naturally occurs and cultivated near commercial orchards. In a recent study carried out in a fragment of an upland forest in the south of the state of Amapá, Sousa et al. (2021) estimated that 1 ha of *Geissospermum argenteum* Woodson (Apocynaceae) forest can host an average of 2,500 parasitoids of four species. In this reservoir plant, parasitism is generally less than 10%, but this is compensated by the high rate of infestation by fruit flies of no economic importance and by the diversity of associated generalist parasitoids. This study highlights the importance of conservation of native habitats, as well as restoration and planting of host plant species in areas close to commercial orchards.

Another amazonian tree species, *Spondias mombin* L. (Anacardiaceae), has also shown considerable potential as a fruit fly parasitoid reservoir plant, with reported parasitism rates of up to 40% of fruit fly puparia (Cunha et al., 2011; Deus et al., 2013; Sousa et al., 2014; Adaime, 2016; Almeida et al., 2016). From both an economic and biological control perspective it would be valuable to evaluate the potential of *S. mombin* to serve as a parasitoid reservoir and, by extension, contribute to biological control of tephritids in the region (Deus & Adaime, 2013; Adaime, Lima & Sousa, 2018).

In this review we discuss the potential of *S. mombin* as a reservoir plant for fruit fly parasitoids based on a unique long-term data set from the northern Brazilian state of Amapá in the far east of Amazon. For nearly two decades researchers at the Embrapa Amapá and Federal University of Amapá have collected information on *S. mombin* from 12 of 16 municipalities in the state. Given the scarcity of research on ecosystem services provided by insects in Brazil, particularly in natural environments and in the Amazon Biome (details in Ramos et al., 2020), this review will be particularly useful for researchers and students working on host plants and the biological control of fruit flies. We also argue for the importance of conserving *S. mombin* in its natural environment on the basis of its role in maintaining populations of fruit fly parasitoids.

### Characterization of the study area

**Figure 1:**
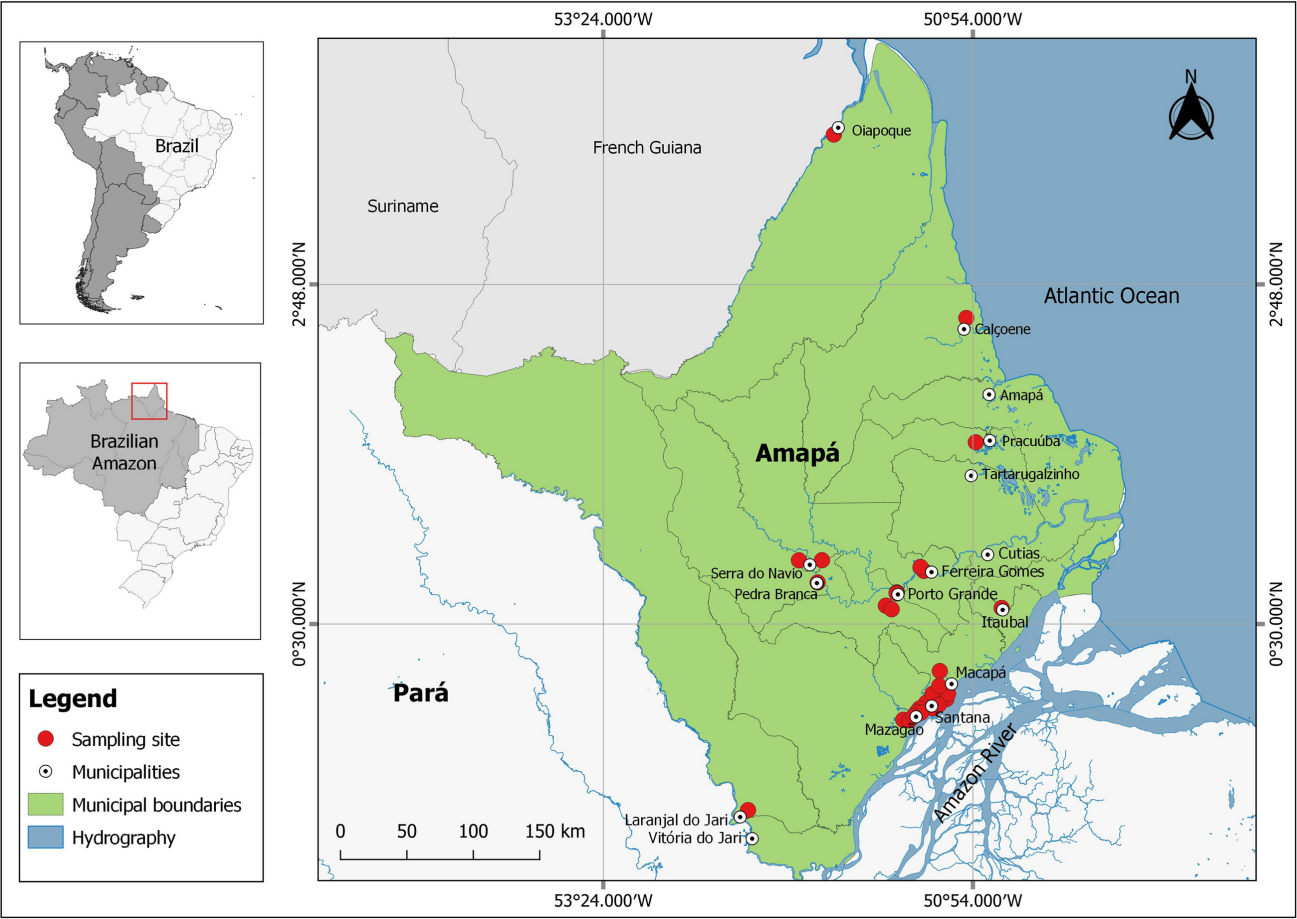
Sampling sites for *Spondias mombin* fruits in several municipalities of the state of Amapá, Brazil.

The state of Amapá (143,453.70 km^2^), located in the far north of Brazil (Fig. 1) is considered the most preserved state in Brazil with approximately 72% of its territory under some form of environmental protection (Conservation International Brazil, 2007). It thus constitutes an excellent case study for investigating natural relationships between native fruits, tephritid species and associated parasitoids.

The state is bordered to the south and west by the State of Pará, to the east by the Atlantic Ocean, to the north by French Guiana and to the northwest by Suriname (Porto, 2007). The climate, according to the Koëppen-Geiger classification, is of the Aw (tropical savanna) and Am (tropical monsoon) type, with an average annual temperature of 26 °C and an average annual precipitation between 2,300 and 2,400 mm (Peel, Finlayson & McMahon, 2007). The rainy season occurs from January to June and a characteristically dry period is more frequent from September to November (IBGE - Instituto Brasileiro de Geografia e Estatística, 2021). The soils are of the type Yellow Latosol, Red-Yellow Latosol, Red-Yellow Argisol and Gleissolos (Alves, Alves & Mochiutti, 1992). The Amapá ecosystems, due to their super humid equatorial climate, present themselves in the form of forests, very rich within their plant variety, and is divided into: upland forest (not affected by floods), floodplain forest (flooded only during the flooding of rivers), and fields (flooded fields, closed fields and clean fields) (Morais, 2000; Zoneamento Ecológico Econômico, 2008).

### The study species *Spondias mombin* L. (Anacardiaceae)

The genus *Spondias* (Anacardiaceae) includes 18 species distributed in the Neotropics, Asia and Oceania (Mitchell & Daly, 1995). *Spondias mombin* (the ‘yellow mombin’ or ‘hog plum tree’), popularly known in Brazil as the “taperebá” or “cajá” tree, is found in the both the Atlantic Forest and Amazon in upland and floodplain forest environments, and is also present in inhabited areas, albeit in a sub-spontaneous state (Cavalcante, 2010). It is an economically important species in the North and Northeast states of Brazil where it is cultivated at a small scale. Its fruit can be consumed *in natura* or processed into pulps, juices, jams, nectars and ice creams of excellent quality and high commercial value. The species has only recently been commercially exploited and, as yet, there is insufficient technical knowledge for the development of large-scale enterprises (Sacramento & Souza, 2009).

*Spondias mombin* is a large tree, able to grow to heights of over 25 m which mainly fruits during the rainy season (Queiroz, 2000). In Amapá, the flowering peak of *S. mombin* occurs from September to October and fruiting lasts approximately eight months, peaking from November to March (Freitas, Santos & Oliveira, 2010). The fruits are globose or elliptical drupes, ranging in color from yellow to light orange, with a thin, smooth skin, juicy pulp and tangy-sweet flavor. They are consumed both in natura or processed into juices and ice creams that are sold in farmer’s markets in the North and Northeast states of Brazil (Santos-Serejo et al., 2009; Cavalcante, 2010).

In the Amazon, the fruits are extracted directly from the forest and, to date, there have been very few attempts to develop commercial orchards. The small production volume means that companies that process this fruit are completely dependent on supply from small-scale extractivism, which is seasonal and often insufficient for the viable operation of processing facilities (Souza, 2005). One potential income-generating option for local rural workers would be to set up domestic orchards (Deus, Sousa & Adaime, 2016). *Spondias mombin* is also a potentially good option as a component of Agro-Forest Systems, including those developed for restoring degraded areas (Bezerra, Barros Neto & Silva, 2010).

**Table 1:** Occurrence of fruit fly species and their associated parasitoids in *Spondias mombin* in the state of Amapá, Brazil.

Municipalities	SC/SI^a^	Fruits (n)	Mass (kg)	Puparia (n)	*Anastrepha* spp. + Parasitoids^b^	Infestation (puparia/kg)	PP^c^ (%)	References^d^
Macapá	6/6	8,032	93.44	2,335	Ao, Aa + Da, Ob^e^	25.0	11.6	Silva, Jesus & Silva (2006a)
Ferreira Gomes	8/7	924	8.98	470	Ao, Aa + Da, Ob^e^, Ua	52.3	21.7	Silva & Silva (2007)
Itaubal do Piririm	5/5	673	6.28	886	Ao, Aa, As + Da, Aan	141.1	11.9	Silva et al. (2007b)
Santana	6/6	794	8.52	389	Ao + Da, Ob^e^, Aan	45.7	10.5	Silva et al. (2007a)
Serra do Navio	5/5	1,374	16.46	1,178	Aa, Ao, Aso, Ast + Aan, Da, Ob, Ua	71.57	5.85	Deus et al. (2009)
Pedra Branca do Amapari	6/5	1,485	19.61	413	Aa, Ao, Ast + Aan, Ob	21.1	3.38	Deus et al. (2009)
Macapá	1/1	75	0.95	135	Ao + Da, Ob	141.7	46.6	Cunha et al. (2011)
Laranjal do Jari	3/3	1,480	12.00	630	Ao, Aa + Da, Aan, Ob, Ua	58.0	5.3	Silva et al. (2011b)
Macapá	4/4	267	2.30	149	Ao, Aa + Da, Ob	64.7	46.9	Deus et al. (2013)
Porto Grande	4/4	216	3.20	157	Ao, Aa + Ob	49.1	3.18	Deus et al. (2013)
Pracuúba	1/1	100	0.82	287	Ao + Aan	349.6	1.74	Deus et al. (2013)
Serra do Navio	1/1	48	0.83	320	Ao + Ua, Ob, Ap	385.1	5.0	Deus et al. (2013)
Ferreira Gomes	3/3	45	0.82	35	Ao, Aa + Da, Ob	42.7	17.2	Sousa et al. (2014)
Macapá	3/2	45	0.43	5	Ao + Da, Ob	11.6	40.0	Sousa et al. (2014)
Mazagão	2/2	30	0.41	34	Ao, Ast, Aa, Af + Da	82.9	20.6	Sousa et al. (2014)
Porto Grande	3/2	45	0.38	37	Ao, Aa + Ob	97.4	18.9	Sousa et al. (2014)
Macapá + Santana	30/20	600	6.7	298	Ao, Aa + Da, Ob	44.48	27.8	Nascimento et al. (2015)
Santana	11/11	387	4.9	853	Ao, Af, Aa, Bc + Da, Ob, Ua	174.1	43.1	Almeida et al. (2016)
Mazagão	10/9	1,036	10.85	734	Aa, Ao, Af + Ob, Da, Aan, Ua, Ap	66.8	18.9	Sousa et al. (2016)
Porto Grande	10/10	1,204	12.59	1,232	Ao, Aa, Ast + Ob, Da, Aan, Ua	99.8	9.6	Sousa et al. (2016)
Oiapoque	10/9	1,045	13.63	749	Ao, Aa + Ua, Aan, Ob	56.8	2.5	Sousa et al. (2016)
Calçoene	3/3	51	0.67	41	Ao + Ua	61.2	4.9	Adaime et al. (2017)
Mazagão	3/3	45	0.37	83	Ao, Aa + Ob, Da	224.3	22.9	Lemos et al. (2017)
Porto Grande	6/6	90	1.32	600	Ao, Aa, Af, Ast + Ob, Da, Aan, Ap, Ua	454.5	14.3	Lemos et al. (2017)
Santana	5/5	75	0.61	235	Ao, An + Ob, Da	385.2	17.0	Lemos et al. (2017)
Macapá	19/16	2,937	30.3	1,343	Ao, Aa, Ast, Af + Da, Ob, Aan, Ua	44.3	25.7	R Adaime, 2007 (unpublished data)
Mazagão	28/21	2,891	32.6	826	Ao, Aa, Ast + Da, Ob	25.3	23.5	R Adaime, 2007 (unpublished data)
Porto Grande	10/9	632	9.9	82	Ao, Aa + Ob, Da, Ap, Aan	83.2	12.9	R Adaime, 2007 (unpublished data)
Santana	27/25	2,973	26.8	1,039	Ao, Aa, Af + Da, Ob, Ap, Aan, Ua	38.8	17.5	R Adaime, 2007 (unpublished data)
Macapá	10/9	373	6.95	565	Ao, Aa + Da, Ob, Ua	81.3	10.3	E Deus, 2008 (unpublished data)
Mazagão	2/2	123	1.43	48	Ao, Aa + Da, Ob, Aan, Ua	33.5	31.3	E Deus, 2008 (unpublished data)
Porto Grande	2/2	54	0.78	338	Ao, Aa + Ob, Aan	431.6	5.3	E Deus, 2008 (unpublished data)
Santana	5/5	269	2.77	66	Ao + Da	23.8	4.5	E Deus, 2008 (unpublished data)
**TOTAL**	**252/223**	**30.418**	**338.60**	**16,592**	–	–	–	–

**Notes.**

a SC/SI: samples collected/samples infested.

b Descending order of abundance

c Average parasitism percentage

d Chronological order of publication

e Cited in the original works as *Opius* sp.

Aa *Anastrepha antunesi* Af *Anastrepha fraterculus* Ao *Anastrepha obliqua* Aso *Anastrepha sororcula* Ast *Anastrepha striata* Bc *Bactrocera carambolae + Aan = Asobara anastrephae* Ap *Aganaspis pelleranoi* Da *Doryctobracon areolatus* Ob *Opius bellus* Ua *Utetes anastrephae*

### Fruit fly infestation of *S. mombin* in Amapá

Studies on fruit flies and parasitoids in Amapá have resulted in 14 publications based on 103 samples. Data from another 119 samples are available in unpublished form (Table 1). For the present analysis we have collated data from 252 samples, representing 30,418 fruits (338.60 kg) from *S. mombin*, of which 223 (88.5%) were infested by fruit flies (Table 1). In the most intensive work carried out, the vast majority of samples were collected from January to May, the rainy season and the greatest abundance of ripe fruits in the field (R Adaime, 2007, unpublished data; Jesus-Barros et al., 2012). However, it is possible to find fruits in the field in the driest months, then some samples were collected in September, November and December (R Adaime, 2007, unpublished data; Sousa et al., 2014). Considering that there is little fruiting during this period, it is necessary to carry out studies to evaluate the role of these fruits in maintaining populations of fruit flies and their parasitoids in native and agricultural areas.

From the collected fruits we have obtained 16,592 puparia of fruit flies with infestation rates generally lower than 100 puparia/kg of fruit; the highest reported being 454.5 puparia/kg (Table 1). These data were collected following two typical methodologies: (i) in most studies (76.9%), a grouped fruits methodology was adopted, whereby the fruits are packaged together, with several fruits from the same plant constituting a single sample; (ii) Cunha et al. (2011), Sousa et al. (2014) and Lemos et al. (2017) used an individualized fruit methodology, where each fruit represents a subsample. When we take together the amount of fruits from an individualized sample, they are equivalent to a grouped sample. Therefore, for the purpose of discussion we consider everything as a “grouped sample”.

Five species of *Anastrepha* have been reported from *S. mombin* fruits in the state of Amapá: *Anastrepha antunesi* Lima, *Anastrepha fraterculus* (Wiedemann), *Anastrepha obliqua* (Macquart), *Anastrepha sororcula* Zucchi, and *Anastrepha striata* Schiner (Table 1). Infestation by the invasive *Bactrocera carambolae* Drew & Hancock (Brasil, 2018) has also been recorded (Silva et al., 2011a; Lemos et al., 2014). Of these species, *A. obliqua* is the most commonly found on *S. mombin* fruits (Silva, Lemos & Zucchi, 2011; Deus, Sousa & Adaime, 2016), although *A. antunesi* is also frequently obtained from samples, albeit at a lower abundance than *A. obliqua*.

In Brazil, 121 species of *Anastrepha* have been recorded (Zucchi & Moraes, 2021). However, only a few of these species are economically important, including *A. fraterculus* (the South American fruit fly), *A. obliqua* (the West Indian fruit fly), and *A. striata* (the guava fruit fly) (Leonardo et al., 2017). These species were obtained from several samples of *S. mombin* collected in Amapá (Table 1). Exactly 116 host plants of *A. fraterculus*, 51 of *A. obliqua* and 31 of *A. striata* have already been reported in Brazil (Zucchi & Moraes, 2021; Adaime et al., 2014). *Anastrepha antunesi* and *Anastrepha sororcula* have no economic importance (Uramoto & Zucchi, 2009), the former being frequently associated with *S. mombin* and the latter with *Psidium guajava* (Adaime, Sousa & Pereira, 2016). *Bactrocera carambolae* (the carambola fruit fly), native to Asia, is an invading species in South America (Castilho et al., 2019a). Its first detection in Brazil was in 1996, in Oiapoque county, state of Amapá (Silva et al., 2004). This species is currently classified in Brazil as a quarantine pest and is under official control by the Ministry of Agriculture and Food Supply (Brasil, 2018). In addition to *S. mombin*, it has been recorded on 25 other host plants in Brazil, all of which occur in Amapá (Belo et al., 2020).

Although it is possible to find fruits of *S. mombin* with significant infestation (up to 8 pupae), the simultaneous occurrence of *A. obliqua* and *A. antunesi* is uncommon (only 0.5 to 1.3% of the fruits), which may mean low interspecific competition (Cunha et al., 2011; Sousa et al., 2014; Nascimento et al., 2015). Another factor that may explain the rare sharing of the same fruit by *A. obliqua* and *A. antunesi* is the significant abundance of this plant species in the sampling areas (Nascimento et al., 2015) - in addition to the presence of fruits from other hosts, since they are polyphagous (Zucchi & Moraes, 2021). This highlights the importance of studies based on individual fruits, since these allow researchers to better study the tritrophic relationship between the host fruit, the larvae of flies that infest it, and the parasitoids associated with them (Silva et al., 2011c).

### Fruit fly parasitoids from *S. mombin* in Amapá

The vast majority of fruit samples of *S. mombin* contained parasitoids of fruit flies, especially those collected in the rainy season (Table 1). Four species of Braconidae have been recorded in association with *S. mombin* in the state of Amapá: *Doryctobracon areolatus* (Szépligeti), *Opius bellus* Gahan, *Asobara anastrephae* (Muesebeck), and *Utetes anastrephae* (Viereck). The eucoiline parasitoid *Aganaspis pelleranoi* (Brèthes) Figitidae has also been reported on *S. mombin*. *Doryctobracon areolatus* and *O. bellus* are the most widely distributed species in Brazil and the most common in fruits from the Amazon region (Silva, Lemos & Zucchi, 2011; Sousa, Adaime & Pereira, 2016). *Asobara anastrephae*, *U. anastrephae*, and *A. pelleranoi* are also abundant in the region, but are generally represented by a few specimens in the samples (Deus & Adaime, 2013; Sousa, Adaime & Pereira, 2016).

Braconidae parasitoids are the most important natural enemies of fruit flies at the global level (Wharton, 1996; Ovruski et al., 2000), besides being widely distributed. They are frequently obtained from fruits infested by fruit flies in the Brazilian Amazon and in Brazil as a whole (Canal & Zucchi, 2000; Sousa, Adaime & Pereira, 2016). The Figitidae occur throughout Brazil, although they are poorly studied (Guimarães & Zucchi, 2011). In the Brazilian Amazon, there are records in the states of Amazonas, Amapá, Pará and Roraima (Sousa, Adaime & Pereira, 2016).

Members of the Braconidae and Figitidae are mainly koinobiont endoparasitoids that oviposit in the larval stage, developing in the living host and killing it at the end of the cycle when the adult wasp emerges from the host’s puparium (Ovruski et al., 2000; Brodeur & Boivin, 2004; Marinho, Costa & Zucchi, 2018). Most native Braconid parasitoids are generalists, being able to parasitize *Anastrepha* species on a wide variety of plant species (López, Aluja & Sivinski, 1999). They are the most studied fruit fly parasitoids worldwide (Wharton, Marsh & Sharky, 1997), being preferred in biological control programs due to host specificity by the Tephritidae family (Clausen, 1940). Of the four species of Figitidae that occur in the Brazilian Amazon, *A. pelleranoi* is the most generalist, being frequently associated with Tephritidae puparia (Guimarães & Zucchi, 2011).

Parasitism rates varied between studies, reaching as high as ∼50% of puparia (Table 1). Specifically, Cunha et al. (2011) recorded a parasitism rate of 46.6%, almost equally split between *D. areolatus* (57.1% of specimens) and *O. bellus* (42.9%). Nascimento et al. (2015) reported a somewhat lower parasitism rate of 27.8% for the same species of parasitoids [*D. areolatus* (55.4%) and *O. bellus* (44.6%)]. Overall, the braconid *D. areolatus* is the most abundant species in samples of *S. mombin* in Amapá, including the capital city Macapá (Silva, Jesus & Silva, 2006a) and Santana (Silva et al., 2007a). However, in some samples *O. bellus* was the most abundant species Deus et al., (2013). In the Brazilian Amazon, *D. areolatus* and *O. bellus* have been associated with 16 and nine species of *Anastrepha*, respectively (Marinho, Silva & Zucchi, 2011). The association of a given parasitoid species with a fruit fly species can only be considered when they are found exclusively (one species of parasitoid and one fruit fly species) in a sample of a single fruit species (Leonel Junior, Zucchi & Wharton, 1995). Under these criteria, the parasitoids *D. areolatus* and *O. bellus* are associated with *A. obliqua* (Sousa et al., 2014) and *A. antunesi* (Nascimento et al., 2015) in S. mombin fruit.

Although braconid parasitoids prefer certain species of host flies, the physical (weight, color and size) and chemical (chemical volatiles) characteristics of fruit are also important factors in parasitoid attraction (Marinho, 2009). For example, parasitism is more difficult in large fruits, as the larvae have to burrow deep into the pulp (Sivinski, Aluja & López, 1997). Consequently, fruit weight and size are usually inversely proportional to the rate of parasitism, i.e., the heavier and larger the fruit, the less likely it is to be parasitized (Hernández-Ortíz, Pérez-Alonso & Wharton, 1994; López, Aluja & Sivinski, 1999). Fruits of *S. mombin* are quite variable in size (Cunha et al., 2011): mean smaller diameter 26.5 ± 0.24 mm (ranging from 21.4 to 33.7), mean larger diameter 36.2 ± 0.30 mm (ranging from 29.3 to 43.5), weight mean 12.7 ± 0.31 g (ranging from 6.6 to 22.7) and pulp thickness mean 9.5 ± 0.14 mm (ranging from 6.7 to 12.2). Cunha et al. (2011) found no significant correlation between percentage of parasitism and fruit weight, with no correlation between parasitism and pulp thickness. This suggests that the range thickness of *S. mombin* fruits does not strongly influence parasitism or the efficiency of parasitoids in finding larvae of their host flies. Significant differences in the chemical composition of fruits of *S. mombin* were observed by Bezerra, Barros Neto & Silva (2010). The authors collected only two samples from the municipality of Mazagão, state of Amapá, and observed different patterns for almost every qualitative parameter that was analyzed, highlighting the enormous diversity of genetic material in the region. To date, no studies have been published showing the relationship between parasitism and chemical characteristics of *S. mombin* fruits.

Ovipositor length also varies among species (*D. areolatus* −3.15 to 4.59 mm; *O. bellus* −0.93 to 1.66 mm; *U. anastrephae* −0.85 to 1.87 mm; and *A. anastrephae* −2.49 to 3.28 mm (Marinho, 2009) and is a determining factor in parasitism. The shorter ovipositor of *U. anastrephae* may be an adaptation for foraging on smaller fruits, potentially competing with *D*. *areolatus*, which has a longer ovipositor (Sivinski, Aluja & López, 1997).

### Potential for biological control of fruit flies using parasitoids

In Amapá, some forest inventories have already been completed in “várzea” floodplain forest ecosystems, providing phytosociological data on *S. mombin* (Table 2). The highest concentrations of this species are located along the Jari River basin, with densities of 69 to 130 individuals per hectare (Carim et al., 2017).

**Table 2:** Structural data on arboreal vegetation of “várzea” and “igapó” floodplain forests and *Spondias mombin* populations in the state of Amapá.

Location	Forest structure				*Spondias mombin* (individuals/ha)	References
	Inventoried area (ha)	DBH^*^	No. of ind.^**^	No. of sp.^***^		
High “Várzea” Floodplain (Rio Preto to Bailique)	1.0	≥5 cm	4,244	104	19	Queiroz (2004)
Low “Várzea” Floodplain (Rio Preto to Bailique)	1.0	≥5 cm	4,635	98	58	Queiroz (2004)
Rio Mazagão and Mazagão Velho	5.0	≥10 cm	2,068	82	12	Carim, Jardim & Medeiros (2008)
Mazagão	0.5	≥5 cm	9,618	111	10	Farias (2012)
Laranjal do Jari (“Igapó” Forest)	13.0	≥10 cm	5,114	285	130	Carim et al. (2017)
Mazagão (“Várzea” Floodplain)	13.0	≥10 cm	5,461	98	69	Carim et al. (2017)

**Notes.**

* DBH = Diameter at Breast Height.

** No. of ind. = number of individuals of all species inventoried in the area.

*** No. of sp. = number of species inventoried in the area.

Considering fruit infestation rates by fruit flies and fruit fly parasitism rates by parasitoids, we can make a crude estimate of the number of parasitoids ‘produced’ per 1 kg of *S. mombin* fruits. Specifically, our studies suggest that floodplain forest ecosystems in the state of Amapá can ‘produce’ up to 66.7 specimens of parasitoids of *Anastrepha* from 1 kg of fruits of *S. mombin* (Table 1). If we consider that 10 to 130 *S. mombin* trees can be found per hectare in a floodplain forest (Table 2), and that each tree can potentially produce up to 10,000 (=100 kg) fruits (Janzen, 1985), then these forests conceivably host over 900,000 parasitoids per hectare.

When estimating the production of parasitoids per hectare, we must take into account the natural mortality factors of these insects. In field conditions, the risk of mortality is associated, for example, with the search for a source of food, which, if not found before the insect runs out of energy, will cause its death by starvation (Bernstein & Jervis, 2008). There is also the incidence of extrinsic factors, such as climate (Weisser, Völkl & Hassell, 1997), predation (Jervis, 1990; Heimpel, Rosenheim & Mangel, 1997; Rosenheim, 1998) and desiccation (Jervis, Ferns & Heimpel, 2003). In addition, we must consider that the rates of parasitism in fruits that are collected in the field and placed under laboratory conditions do not accurately represent reality. These fruits, when removed from the natural environment, may contain larvae of first and second instars that are not yet susceptible to being parasitized Adaime et al., (2018). Thus, when the fruits containing the immatures are removed from the field, it is no longer possible for parasitism to occur (Uchôa-Fernandes et al., 2003). Thus, the actual rate of parasitism may be even higher than predicted, as observed by Adaime et al., (2018) in larvae of *A. coronilli* that infest fruits of *B. grossularioides*. Parasitism may also be underestimated in studies using fallen fruit because, when the infested fruits fall, larvae abandon them and penetrate the soil to begin the pupa phase Adaime et al., (2018). This is a limitation for most studies on large canopy trees because in the cases fruit samples are usually collected from the soil. In any case, our estimated amount of parasitoids per hectare of foodplain forest (900,000) is much higher than that estimated by Sousa et al. (2021) for a fragment of upland forest in Amapá, with the occurrence of *G. argenteum*, natural host of *Anastrepha atrigona* Hendel (2,500 parasitoids per ha). Sousa et al. (2021) use a specific plot-based methodology and a robust statistical approach, the application of which to *S. mombin* in Amapá floodplain forests would make it possible to measure the contribution of this plant species to maintaining fruit fly parasitoid populations.

To date, besides Amapá, *S. mombin* has been sampled in other four of the nine states that make up the Brazilian Amazon. The highest percentages of parasitism recorded in each state were 29.5% in Acre (Thomazini & Albuquerque, 2009), 30.0% in Amazonas (Dutra et al., 2013), 31.1% in Roraima (Marsaro Júnior et al., 2010), 46.9% in Amapá and 70.8% in Pará (Brandão et al., 2019) (Table 3). The density of parasitoids may be even higher than estimated for Amapá (66.7 parasitoids/kg of fruit): Marsaro Júnior et al. (2010) obtained 165 parasitoids/kg of fruit from Roraima (also in the Brazilian Amazon), while López, Aluja & Sivinski (1999) obtained an impressive 207 parasitoids/kg of *S. mombin* fruit in Mexico. Also in Mexico, Murillo et al. (2015), found that 88% of the *A. obliqua* larvae obtained from *S*. *mombin* were parasitized. Due to the great difference in sampling effort, it is not possible to directly compare the data collected in Amapá during almost 20 years (Table 1) with the point data produced in other locations in the Brazilian Amazon (Table 3) and in Mexico. Therefore, it is necessary that studies be carried out on a larger spatial and temporal scale to identify which factors influence the percentage of parasitism and the number of parasitoids obtained per kilo of fruit.

**Table 3:** Ocurrence of parasitoids in fruits of *Spondias mombin* infested by *Anastrepha* spp. in the Brazilian Amazon.

States Municipalities	SC/SI^a^	Fruits (n)	Mass (kg)	Puparia (n)	*Anastrepha* spp. + Parasitoids	Infestation (puparia/kg)	PP^b^ (%)	References
**Acre**								
Bujari	1/1	271	2.40	468	Ao + Ob, Da, Ua	195.0	29.5	Thomazini & Albuquerque (2009)
**Amazonas**								
Manaus	ni	1,038	7.9	1,491	Ao + Da, Ob, Ua	188.7	9.2	Dutra et al. (2013)
Presidente Figueiredo	ni	232	3.1	586	Ao, Aa + Ob, Aan, Da, Ua, Ap, An	189.0	30.0	Dutra et al. (2013)
**Pará**								
Afuá	ni	6,130	53	636	Ao, Aa + Da, Ob^c^	12.0	27.0	Silva, Jesus & Silva (2006b)
Belém	ni	ni	ni	413	Ao, Aa + Da, Ob^c^	ni	8.7	Castilho, Lemos & Oliveira (2008)
Belém	2/2	100	1.20	560	Ao, Af, Aa + Da, Ob	466.7	52.7	Castilho et al. (2019b)
Belém	10/10	3,770	27.0	896	Ao, Aa, Af + Da, Ob	33.2	6.7^d^	Brandão et al. (2019)
**Roraima**								
Amajari	1/1	30	0.54	286	Ao, Aa + Ob, Ua, Da	532.6	31.1	Marsaro Júnior et al. (2010)
Boa Vista	10/10	268	2.21	543	Ao + Ob, Ap	245.7	13.1	Marsaro Júnior et al. (2011)
Pacaraima	2/2	85	1.11	150	Ao, Aa + Ob, Ua	135.1	7.3	Marsaro Júnior et al. (2011)
Bonfim	4/4	90	0.73	252	Ao + Ob, Da, Ua	345.2	10.7	Marsaro Júnior et al. (2011)
Normandia	1/1	10	0.05	7	Ao + Da	140.0	14.3	Marsaro Júnior et al. (2011)
Cantá	2/2	73	0.82	165	Ao, Ast + Ob	201.2	9.1	Marsaro Júnior et al. (2011)

**Notes.**

a SC/SI: samples collected/samples infested, PP: average percentage of parasitism, ni: not informed at work.

b Average parasitism percentage

c Cited in the original works as *Opius* sp.

d In one sample the parasitism reached 70.8%.

Ao Anastrepha obliqua Aa *Anastrepha antunesi* Af *Anastrepha fraterculus* Ast *Anastrepha striata + Aan = Asobara anastrephae* Da *Doryctobracon areolatus* Ob *Opius bellus* Ua *Utetes anastrephae* Ap *Aganaspis pelleranoi* An *Aganaspis nordlanderi*

We should also take into account that *S. mombin* does not occur exclusively in floodplain forests, but can also be found in upland forest and in secondary vegetation areas where it regenerates spontaneously from seeds or from stakes and roots (Bosco et al., 2000). Amapá has a total floodplain forest area of approximately 543,348 hectares and 10,362,374 hectares of upland forest (Zoneamento Ecológico Econômico, 2008), in addition to highly conserved upland forests. Another factor to be considered is that the majority of Amapá floodplain forests are difficult to access. This undoubtedly contributes to conservation of the region, but also hampers data collection leading to incomplete knowledge about many aspects of biodiversity (Gaston & Rodrigues, 2003; Mace, 2004; Ladle & Hortal, 2013).

We have shown that the floodplain forests and upland ecosystems of Amapá have enormous potential as areas for the multiplication of parasitoids of fruit fly species. In these ecosystems (where *S. mombin* is present) millions of parasitoids from at least five species (*O*. *bellus*, *D. areolatus*, *A. anastrephae*, *U. anastrephae* and *A. pelleranoi*) are produced every year at zero economic cost. The results of almost 20 years of research indicate that *S. mombin* can potentially be used to maintain and multiply parasitoids which, in turn, act as agents of natural biological control of agricultural pest species in the genus *Anastrepha*. In the Brazilian Amazon, parasitoids from *S. mombin* can regulate fruit fly populations in fruits of socioeconomic importance, for example *Anastrepha striata* in guava (Sousa et al., 2019). In addition to its role as a host for biological control agents, *S*. *mombin* could contribute both economically and environmentally to projects involving the restoration of legal reserves. These reserves are created by large landowners as part of their obligations under federal law for preserving a proportion of natural habitat. The main goals of legal reserves are to ensure an economical and sustainable use of natural resources in rural properties, to help conserve and rehabilitate ecological processes, and to promote the conservation of biodiversity (Brasil, 2012).

Finally, although Amapá is considered the best-preserved state in Brazil (Conservation International Brazil, 2007), large swathes of other states in the Brazilian Amazon are degraded or in the process of degradation. Given this situation, recovering these areas using native plant species with commercial potential and that can also provide valuable ecosystem services is an interesting alternative. As such, *S. mombin* has enormous potential for use in restoration and agroforestry projects.

## Conclusions

Biological control of fruit flies by parasitoids is a potentially important though poorly studied ecosystem service in the tropics. In general, fruit fly parasitoids are generalists (especially Braconidae), and can parasitize larvae of several species of the genus *Anastrepha*, including those considered agricultural pests. Our unique long term data from the Brazilian Amazon clearly demonstrates that the hog plum tree (*Spondias mombin*) is an important reservoir for fruit fly parasitoids. The conservation of the habitats containing this tree is therefore important for the maintenance of the biological control ecosystem service provided by these parasitoids.

Having established the importance of *S. mombin* for maintaining fruit fly parasitoids, additional studies are now needed to better understand fruit fly-parasitoid interactions and to determine the complex relationships between landscape structure and parasitoid population and community dynamics. Such information, especially relating to behavioral patterns of parasitoids, is important for developing effective strategies to maintain and enhance the biological control of fruit flies.

Further studies involving *S. mombin* dispersal patterns in floodplain forest and upland environments will also be important. In floodplain forest areas, hydrochoric dispersal may be highly significant, whereas zoochoric dispersal may be more relevant in upland forest environments. More information on this topic will clarify dispersal patterns of *S. mombin* in different ecosystems, and provide information on its possible contributions as a reservoir of parasitoids in adjacent areas. Likewise, a more precise delineation of the areas of occurrence of *S. mombin* in floodplain and upland forests, generally and in Amapá will also support conservation planning. This information, associated with estimates of the quantity of fruit produced per plant during each production season (i.e., knowledge on the phenology of the species), will allow a more realistic evaluation of the potential of *S. mombin* as a reservoir of natural enemies of fruit flies in the Brazilian Amazon.

Finally, we strongly encourage the scientific community to renew efforts to estimate the contribution of natural ecosystems to maintaining populations of fruit fly parasitoids. Not only does this information provide unambiguous support for the conservation of natural habitats, it also directly contributes to the development of sustainable agricultural practices. We thus recommend that research groups from different regions of the world study native plants in their natural environment, if possible, using long-term ecological studies approaches. In this way significant gains will be obtained in terms of understanding the role of forests in maintaining populations of parasitoids and the complex trophic interactions involved.

## Additional Information and Declarations

### Data Availability

The following information was supplied regarding data availability: The data used in this review are published in the articles listed in References. The articles were accessed from ‘Portal Periódicos CAPES’ (www.periodicos.capes.gov.br) and ‘Base de Dados da Pesquisa Agropecuária’ (http://www.bdpa.cnptia.embrapa.br).
